# Low-level plasticizer exposure and all-cause and cardiovascular disease mortality in the general population

**DOI:** 10.1186/s12940-022-00841-3

**Published:** 2022-03-09

**Authors:** Guowei Zeng, Qi Zhang, Xiaowei Wang, Kai-Hong Wu

**Affiliations:** 1 Department of Cardiothoracic Surgery, Children’s Hospital of Nanjing Medical University, 72 Guangzhou Road, Nanjing, 210008 China; 2 Department of Cardiovascular Surgery, The First Affiliated Hospital of Nanjing Medical University, Nanjing, 210029 China

**Keywords:** phthalate, CVD mortality, NHANES, DEHP, MECPP

## Abstract

**Background:**

Plasticizers, also called phthalates, are a group of chemicals widely used in daily life. A previous report showed no significant association between phthalate metabolite concentrations and mortality. We investigated the association of urinary phthalate levels and individual phthalate metabolite levels with all-cause and cardiovascular disease (CVD) mortality after standardizing the phthalate concentration.

**Methods:**

A total of 6,625 participants were recruited from a nationally representative sample of adults aged 40 years or older who were enrolled in the National Health and Nutrition Examination Survey (NHANES) between 2003 and 2014 and were followed up through December 31, 2015. Data were analyzed from January 2021 to June 2021. NHANES-linked updated National Death Index public access files were used to acquire information on mortality status and cause of death. The present study conducted extended follow-up of an earlier analysis. Cox proportional hazard models were performed to calculate the hazard ratios (HRs) and 95% confidence intervals (CIs) of covariate-adjusted creatinine standardization urinary phthalate concentrations with all-cause and CVD mortality after adjusting for demographics, lifestyle factors and comorbidity variables.

**Results:**

The mean ± standard deviation age of all participants in the final study was 59.9±12.6 years old, and 49.6% of the participants were male. The median follow-up time was 73 months (range 1-157 months). At the censoring date of December 31, 2015, 3,023 participants were identified as deceased (13.4%). A fully adjusted Cox model showed that a urinary di(2-ethylhexyl) phthalate (DEHP) concentration >= 83.4 ng/mL was associated with a slight increase in all-cause mortality (HR 1.27, 95% CI 1.03, 1.57, P for trend= 0.014) and CVD mortality (HR 2.19, 95% CI 1.35, 3.54, P for trend= 0.002). Similarly, urinary mono-2-ethyl-5-carboxypentyl phthalate (MECPP) levels >= 39.2 ng/mL were associated with increased CVD mortality (HR 2.33, 95% CI 1.45, 3.73, P for trend < 0.001). Restricted cubic spline analyses suggested linear associations of DEHP and MECPP levels with all-cause and CVD mortality.

**Conclusion:**

In this large nationally representative sample of American adults, high urinary DEHP and MECPP were significantly associated with all-cause and CVD mortality after adjusting for demographics, lifestyle factors and comorbidity variables.

**Supplementary Information:**

The online version contains supplementary material available at 10.1186/s12940-022-00841-3.

## Introduction

Recent data from the World Health Organization estimate that 2 million lives were lost in 2019 due to exposure to selected chemicals [1]. Over 6000 chemicals are known to be manufactured in commerce globally, while mortality rates are only available for a small number of chemical exposures. As one of the main threats to human health, cardiovascular disease (CVD) is also the main cause of mortality worldwide [2]. In addition, CVD morbidity and mortality increased approximately 1.9-fold (from 271 million to 523 million) and 1.5-fold (from 12.01 million to 18.6 million), respectively, from 1990 to 2019 [2]. In recent years, CVD mortality caused by environmental chemicals has also been a concern. Previous studies have indicated that heavy metals [3–5], fine particulate matter (PM_2.5_) [6, 7], and bisphenol A (BPA) [8] are related to CVD occurrence and CVD mortality. However, there have been few reports about plasticizer additives and CVD mortality.

Phthalic acid esters (PAEs) have been incorporated into plastics as plasticizers to enhance flexibility, pliability, and elasticity to otherwise rigid polymers for the mass production of plastics since the 1940s [9]. Almost all categories of industrial consumer goods contain PAEs, accounting for 40% of the final products [10]. PAEs do not form stable and irreversible covalent bonds with plastics, which makes them easy to leach from the matrix [11]. Consequently, PAEs can be absorbed by the human body after exposure in a variety of ways, including eating, drinking, inhalation and dermal contact [12]. After absorption, these parent compounds are hydrolyzed to their respective biologically active monoesters, which undergo further modification by various oxidation reactions [13]. Finally, both the primary and secondary metabolites can be glucuronidated and then excreted in the urine [14]. Di(2-ethylhexyl) phthalate (DEHP), which has been the most commonly used phthalate for many years, has been substituted, for the most part, in polyvinyl chloride (PVC) materials by di-iso-nonyl phthalate (DiNP) and di-iso-decyl phthalate (DiDP) [10]. DEHP has also been listed at the Annex XIV of Registration, Evaluation, Authorisation and Restriction of Chemicals (REACH) of the European Union since 2015, which means that authorization for specific uses needs to be applied for and approved [15]. However, due to the resistance to chemical, physical and biological degradation of plastics, such chemicals are still widely present in environmental media [9]. Some subsequent epidemiological studies still show that humans are still highly exposed to DEHP [16, 17]. For example, the median values in urine among pregnant women were 15.6 ng/mL in China [18] and 20.4 ng/mL in the United States [19]. In addition to extensive exposure reported, DEHP is still frequently reported to cause potentially crucial health effects recently [20, 21], such as prostate cancer [22], breast cancer [23], coronary heart disease [24], atherosclerosis [25], and insulin resistance [26].

Previous studies found no significant associations between phthalate and CVD mortality [12]. The present study increased the follow-up time and sample size of previous reports by including the updated National Death Index mortality data files. Based on recent literature reports [27], we used standardized adjusted phthalate metabolite data to investigate the associations of DEHP and individual phthalate metabolites with all-cause and CVD mortality by the Cox regression method. In addition, we added the Kaplan–Meier survival analysis curve, restricted cubic spline (RCS) curve and stratification analysis results to examine the association between DEHP and all-cause and CVD mortality.

## Methods

### Study participants

The National Health and Nutrition Examination Survey (NHANES), a survey utilizing a complex, multistage probability sampling design to select a representative sample from the noninstitutionalized civilian population in the United States, is a national cross-sectional survey conducted by the Centers for Disease Control (CDC) National Center for Health Statistics (NCHS). Data from NHANES were collected through interviews, physiological examinations, and laboratory tests and included demographics, lifestyle factors, comorbidity variables and levels of environmental chemicals in biological samples. We obtained publicly available data from NHANES 2003-2014, in which phthalates were measured. Prior to any data collection, consent was obtained from all individuals. The NHANES study protocol was approved by the National Center for Health Statistics Research Ethics Review Board.

### Phthalate assessment

Spot urine specimens were collected at mobile examination centers and shipped on dry ice to the CDC National Center for Environmental Health. The urine samples were stored at −20 °C until analysis. Solid phase extraction coupled with high-performance liquid chromatography–isotope dilution tandem spectrometry was performed to analyze phthalate metabolites by the CDC National Center for Environmental Health laboratory [28, 29]. The phthalate metabolites include mono(carboxynonyl) phthalate (MCNP), mono(carboxyoctyl) phthalate (MCOP), mono-2-ethyl-5-carboxypentyl phthalate (MECPP), mono-n-butyl phthalate (MnBP), mono-(3-carboxypropyl) phthalate (MCPP), mono-ethyl phthalate (MEP), mono-(2-ethyl-5-hydroxyhexyl) phthalate (MEHHP), MEHP, mono-isobutyl phthalate (MiBP), mono-isononyl phthalate (MiNP), and mono-(2-ethyl-5-oxohexyl) phthalate (MEOHP). Table S1 shows the detection rate, median and distribution of phthalate metabolites. The content of these ultimate metabolites in human urine can be used to estimate exposure to the respective parent phthalate that occurred within the previous 24 h [14, 30]. The metabolism of DEHP first involves hydrolysis to MEHP, and then this primary metabolite undergoes several oxidative reactions to form secondary metabolites (MECPP, MEHHP, and MEOHP). Both primary and secondary metabolites can be excreted in urine and feces as free monoester or glucuronide conjugates [13]. Therefore, we summed the concentrations of these four metabolites to create a summary DEHP variable (∑DEHP), as have previous researchers [31].

$$\Sigma DEHP= MEHP+ MECPP+ MEHHP+ MEOHP$$

### Mortality definition

The International Classification of Diseases 10th Revision (ICD-10) codes were used to calculate the leading cause of death. The main outcomes of our study were all-cause mortality and CVD mortality. Any death related to heart disease (I00–I09, I11, I13, I20–I51) or cerebrovascular disease (I60–I69) was regarded as CVD mortality. Death certificate records from the National Death Index were linked to all participants aged 18 years or older in NHANES with a unique sequence number until December 31, 2015 [32].

### Covariates

Covariate information was obtained through a questionnaire survey, body measurement and laboratory testing. Age, sex, race/ethnicity, education level, poverty income ratio (PIR), alcohol consumption, smoking status, and physical activity were recorded by a questionnaire survey. We estimated whether participants had diabetes according to a previous literature report [33]. The specific criteria were as follows: subjects with a self-reported physician diagnosis of diabetes or with no self-reported diabetes diagnosis but A1C levels of 6.5% or higher. Height and weight were measured by body measurement, and then body mass index (BMI) was calculated as follows: weight (kg)/square of height (m^2^). Averages of measured diastolic blood pressure and systolic blood pressure (>140/90 mmHg), self-reported hypertension and antihypertensive therapy use were used to determine whether subjects had hypertension, according to previous studies [34]. Laboratory tests were used to detect total cholesterol, alanine transaminase (ALT) and high-density lipoprotein cholesterol (HDLC) in fasting serum. A Roche Modular P chemistry analyzer was used to measure total cholesterol and HDLC. DxC800 uses a kinetic rate method to measure ALT activity in serum. Demographic (age, sex, and race/ethnicity) and lifestyle factors (education levels, PIR, alcohol consumption, smoking status, physical activity, and BMI) and comorbidities (diabetes, hypertension, total cholesterol, ALT and HDLC) were adjusted as covariates in statistical analysis to reduce the influence of bias.

### Statistical analysis

Continuous variables are displayed as the mean and standard deviation, and categorical variables are displayed as frequencies. The comparisons of continuous variables were performed by the Kruskal–Wallis test because the distribution of continuous data was skewed. The chi-square test was used to compare the categorical variables. In previous studies, urine creatinine was regarded as a covariate or DEHP was divided by urine creatinine, but both of the above methods may result in bias [27]. In this study, the adjusted standardized chemical concentration method was used to quantify the internal exposure of phthalate metabolites in the population. The specific formula is as follows:

Adjusted standardized chemical concentration =urinary phthalate metabolites X (Predicted creatinine value)/(actual creatinine value)

Since these variables (age, sex, race/ethnicity, BMI) can affect observed urine creatinine concentration [35], they were included in the regression analysis for the calculation of the predicted creatinine value, regardless of whether phthalate concentration was detected and mortality was recorded. The ages of subjects range from 20 to 85 years old. For the linear regression fitting equation, the predicted creatinine value was obtained. Cox regression was used to calculate hazard ratios (HRs) and 95% confidence intervals (CIs). The results are presented as crude, model 1 and model 2. Model 1 was adjusted for age, sex, and race/ethnicity. Model 2 was adjusted for the following covariates, age, sex, race/ethnicity, educational levels, PIR, alcohol consumption, smoking status, physical activity, BMI, diabetes, hypertension, total cholesterol, ALT and HDLC, because of their possible influence on phthalate diester exposure or mortality outcomes [12, 36]. The potential dose–response relationships of DEHP and phthalate metabolites with all-cause mortality and CVD mortality were assessed with a restricted cubic spline (RCS) model with three knots [37]. The model was fully adjusted (i.e., covariates from Model 2). We acquired *p* values of nonlinearity by testing the null hypothesis that the estimated value of the second spline equals zero. Kaplan–Meier survival curves were generated to display univariable associations among DEHP and phthalate metabolites and all-cause mortality and CVD mortality. The log-rank test was used to compare the survival times of DEHP and phthalate metabolite quartile groups. As DEHP levels are influenced by covariates, we conducted separate analyses stratified by covariates to calculate the results of Cox regression. The mediation analyses were conducted by using Stata software with the package ‘Paramed’. Kaplan–Meier survival curves and RCS were generated with R software, and the rest of the analyses were performed using Stata software. A *P* value less than 0.05 was considered statistically significant. Holm–Bonferroni correction was considered in the analysis of DEHP metabolites to account for multiple comparisons.

## Results

Table 1 shows the basic information of the groups stratified by DEHP quartiles. The results show that there are significant differences in age, sex, race, education level, exercise, BMI classification and alcohol consumption among people with different urine DEHP concentrations. Specifically, females, individuals who perform moderate or vigorous physical activity, participants who never drink alcohol, and obese subjects presented higher urinary DEHP concentrations, and education level was negatively correlated with DEHP exposure. However, there were no significant differences in PIR, smoking status, total cholesterol, ALT, HDLC, hypertension or diabetes.

**Table 1:** Baseline characteristics of participants in NHANES 2003–2014 by di (2-ethylhexyl) phthalate (DEHP) levels

	Urinary DEHP levels (ng/ml)				*P*
	<27.3	27.3-45.5	45.5-83.4	≥83.4	
Age (years)	59.1±12.3	60.1±12.9	60.3±12.7	59.9±12.6	<0.001
Gender, %					
Male	60.5	48.0	45.7	44.1	<0.001
Race, %					<0.001
Mexican American	10.8	11.9	17.2	19.3	
Other Hispanic	5.6	8.5	7.5	9.0	
Non-Hispanic White	51.8	52.2	47.8	43.4	
Non-Hispanic Black	19.6	20.9	21.4	23.0	
Other Race - Including Multi-Racial	12.3	6.5	6.1	5.4	
Education Level, %					0.007
Less Than 9th Grade	11.4	12.7	16.9	16.7	
9-11th Grade	13.4	15.9	15.7	16.1	
High School Grad/GED or Equivalent	21.6	23.9	24.0	24.7	
Some College or AA degree	27.3	24.3	25.1	25.2	
College Graduate or above	26.3	23.0	18.1	17.2	
Family PIR, %					0.725
<1	15.2	18.1	17.2	15.8	
>=1	76.2	73.1	75.5	75.3	
Missing	8.6	8.8	8.3	8.9	
Physical activity, %					0.010
Never	61.1	57.7	55.6	54.7	
Moderate	21.7	24.3	26.0	26.0	
Vigorous	16.5	16.7	17.0	17.5	
Missing	0.8	1.	1.5	1.8	
Smoking status, %					0.499
Never	4.5	4.1	4.6	4.2	
Former	44.6	43.0	43.5	43.8	
Current	50.9	52.9	51.9	52.1	
Past-year alcohol drinking, %					<0.001
No	23.6	28.4	29.2	30.3	
Yes	68.3	63.8	62.7	62.2	
Missing	8.1	7.8	8.0	7.5	
BMI (kg/m^2^), %					0.013
<25	27.7	27.7	26.4	23.4	
25-30	36.8	32.9	34.6	35.8	
≥30	34.3	38.2	37.3	39.4	
Missing	1.3	1.3	1.7	1.5	
Total cholesterol (mg/dL)	198.9±41.3	198.5±41.5	200.4±39.5	200.0±42.1	0.393
ALT (U/L)	26.4±50.6	24.4±17.5	25.4±15.6	25.3±17.6	0.179
HDLC (mg/dL)	52.5±16.6	53.3±14.9	53.7±16.0	53.8±15.4	0.632
Hypertension, %					0.350
Yes	47.1	48.0	48.1	48.0	
Diabetes, %					0.109
Yes	17.7	17.7	19.0	18.3	

*DEHP* di (2-ethylhexyl) phthalate, *BMI* Body mass index, *ALT* alanine transaminase, *HDLC* high density liptein cholesterol

Table 2 shows the HR and 95% CI of urinary DEHP for all-cause and CVD mortality. Regarding all-cause mortality, DEHP and all-cause mortality were positively associated in both model 1 and model 2. Similarly, all of the associations were positive between DEHP and CVD mortality. In the fully adjusted model, the HR and 95% CI of urinary DEHP concentration for all-cause and CVD mortality were 1.27 (1.03, 1.57) and 2.19 (1.35, 3.54), respectively. When creatinine correction was performed by considering urinary creatinine as a covariate rather than using the standardized chemical concentration, the results were consistent with the above results (Table S2). Table S3 and Table S4 show the results of individual phthalate metabolites and CVD and all-cause mortality, respectively. After Holm–Bonferroni correction, the results showed a positive association between MECPP and CVD mortality (HR 2.33, 95% CI 1.45, 3.73, P for trend < 0.001), and the residual phthalate metabolites were not significantly different.

**Table 2:** The association of urinary Di (2-ethylhexyl) phthalate (DEHP) concentration with all-cause mortality and cardiovascular mortality in NHANES 2003–2014

	Urinary DEHP levels (ng/ml)				P for trend
	<27.3	27.3-45.5	45.5-83.4	≥83.4	
All-cause mortality					
Crude	Ref	1.11 (0.89, 1.37)	1.14 (0.93, 1.41)	1.11 (0.91, 1.36)	0.369
Model 1	Ref	1.11 (0.89, 1.37)	1.19 (0.97, 1.47)	1.31 (1.07, 1.61)	0.005
Model 2	Ref	1.09 (0.88, 1.35)	1.19 (0.96, 1.46)	**1.27 (1.03, 1.57)**	0.014
CVD mortality					
Crude	Ref	1.69 (1.03, 2.78)	1.65 (1.01, 2.69)	1.96 (1.22, 2.69)	0.012
Model 1	Ref	1.64 (1.00, 2.70)	1.66 (1.02, 2.70)	2.25 (1.40, 3.61)	0.001
Model 2	Ref	1.65 (1.00, 2.73)	**1.73 (1.05, 2.83)**	**2.19 (1.35, 3.54)**	0.002

Values are hazard ratio (95% confidence interval).

Crude was not adjusted.

Model 1: adjusted for age (years, continuous), sex (female or male), and race/ethnicity (non-Hispanic white, black, Hispanic-Mexican, or other).

Mode 2 : model 1 plus adjusted for education levels (Less Than 9th Grade, 9-11th Grade, High School Grad/GED or Equivalent, Some College or AA degree, College Graduate or above), poverty to income ratio (<1, ≥1, or missing), physical activity (never, moderate, vigorous or missing), smoking status (never, ever or current), past-year alcohol drinking (no, yes, or missing), body mass index (<25, 25–30, or ≥30 kg/m^2^), total cholesterol (mg/dL, continuous), alanine aminotransferase (U/L, continuous), high-density lipoprotein cholesterol (mg/dL, continuous), hypertension (no/yes), diabetes (no/yes)

**Fig. 1:**
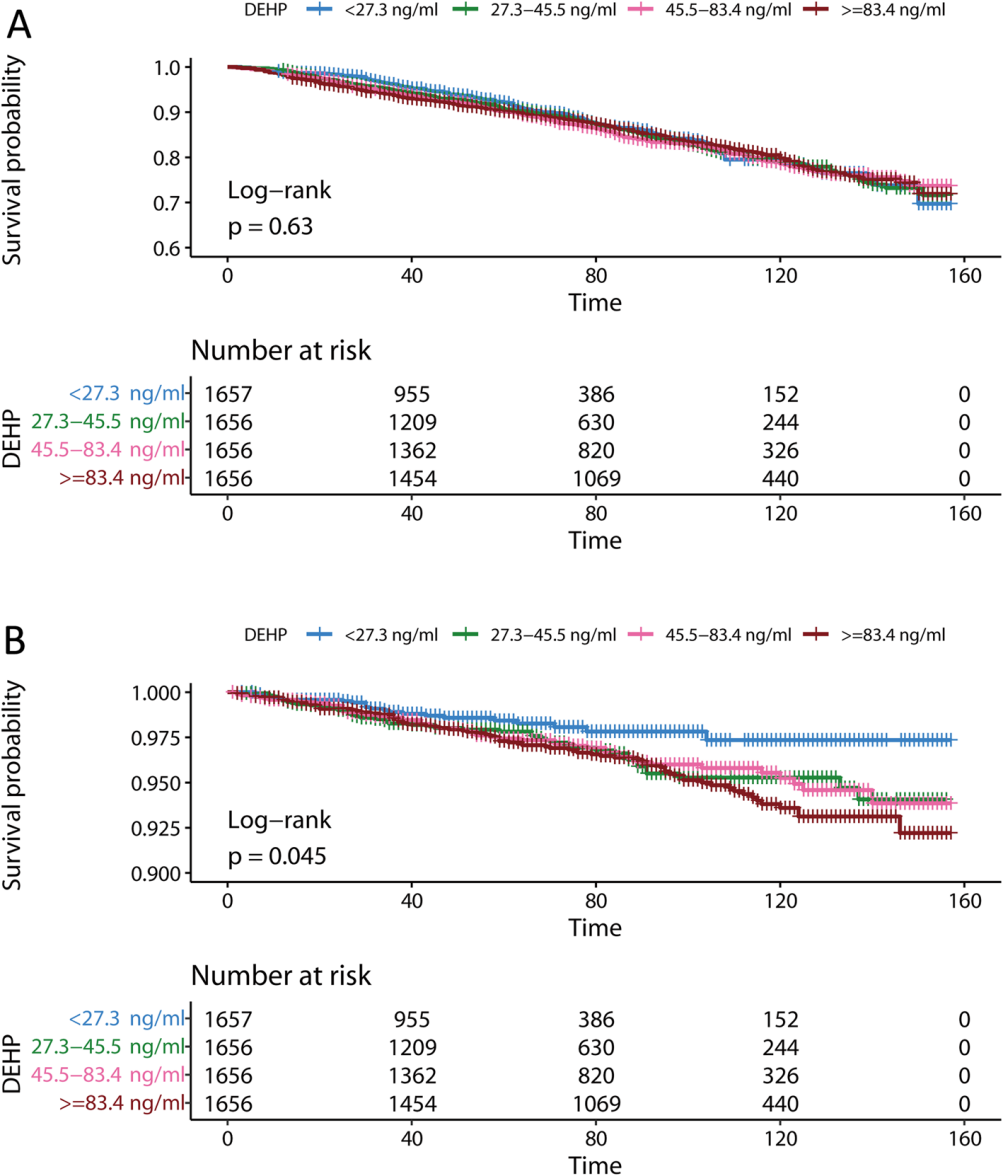
Kaplan–Meier survival curves for DEHP and all-cause and cardiovascular disease mortality. DEHP quartiles were used, in which 4 DEHP concentration groups were assessed for all-cause (**A**) and cardiovascular disease mortality (**B**): lowest (< 27.3 ng/mL), lower (>=27.3 to <45.5 ng/mL), higher (>=45.5 to 83.4 ng/mL) and highest (>=83.4 ng/mL)

Further results of Kaplan–Meier analysis suggested that the all-cause and CVD survival rates decreased with increasing urinary DEHP concentration. The subjects in the highest quartile of urinary DEHP concentration exhibited only increased CVD mortality, and the log-rank *p* values of all-cause and CVD mortality were 0.63 and 0.045, respectively (Fig. 1). Since there was a significant difference in the association of MECPP with CVD mortality in Cox regression, we further assessed the Kaplan–Meier results of MECPP for all-cause and CVD mortality (Figure S1). The results show that the log-rank *p* values were 0.39 and 0.0043, respectively.

**Fig. 2:**
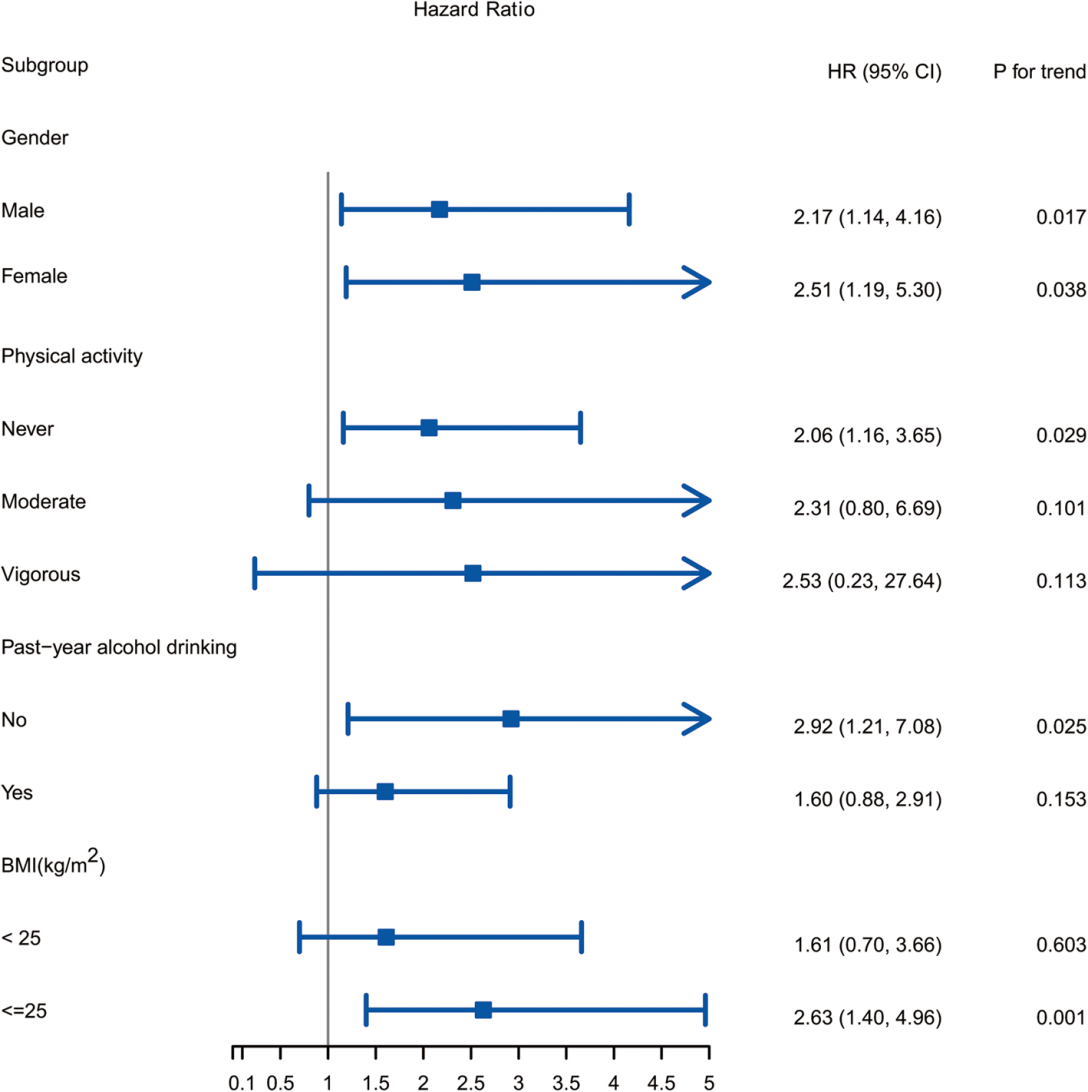
Hazard ratio (95% CI) for cardiovascular disease mortality associated with urinary DEHP adjusted for covariates. Adjustments included demographic (age, sex, and race/ethnicity) and lifestyle factors (education levels, poverty income ratio, alcohol consumption, smoking status, physical activity, and body mass index) and comorbidities (diabetes, hypertension, total cholesterol, alanine transaminase and high-density lipoprotein cholesterol)

According to the results of the stratified analysis shown in Fig. 2, there were significant differences between DEHP and CVD mortality in both males (HR= 2.17, 95% CI 1.14, 4.16) and females (HR= 2.51, 95% CI 1.19, 5.30), and females had a higher risk (higher HR value). In addition, associations between DEHP and CVD mortality were more likely to be observed in people who were never physically active, who never drink alcohol and who had a BMI greater than 25. The results of mediation analysis (Table S5) showed that sex, BMI and physical activity mediated 1.91%, 2.43% and 2.82% association between DEHP and CVD mortality, respectively, with *P* values of 0.001, < 0.001 and < 0.001, respectively. However, the estimated indirect effect of alcohol drinking status was not significant.

Significant positive correlations were identified between DEHP and all-cause and CVD mortality (Figure S2), with no evidence of nonlinear association between DEHP and all-cause or CVD mortality (P nonlinearity = 0.995 and 0.269, respectively). Figure S3 suggests that the MECPP results were similar to those of DEHP (P nonlinearity = 0.531 for all-cause mortality and P nonlinearity = 0.572 for CVD mortality, respectively), and we modeled the linear dose–response associations with the RCS method.

## Discussion

We found for the first time that DEHP and MECPP were positively associated with all-cause and CVD mortality in the general population. In crude and fully adjusted models, DEHP and MECPP were both significantly associated with CVD mortality according to Kaplan–Meier and Cox regression results. Additionally, RCS showed linear relationships of DEHP and MECPP with all-cause and CVD mortality. Subgroup analysis showed that females, individuals who never engaged in physical activity, individuals who did not drink alcohol, and individuals with BMI values greater than 25 were more likely to exhibit an association of DEHP with all-cause and CVD mortality.

A previous study found no significant association between individual DEHP metabolites and CVD mortality [12]. There are three main differences between our study and the abovementioned study. The first point is that the previous study considered urinary creatinine as a covariate. Disease risk factors also affect creatinine concentrations, which may lead to biased results in causal scenarios [38]. We show the results of standard adjustment of urinary creatinine according to a previous article [27], but we still present the results after considering urinary creatinine as a covariate. The second point is that a previous study used the mortality data of 2011, while we used the mortality data of the National Death Index files updated in 2015. The number of all-cause deaths and CVD deaths was greater, and the follow-up time was correspondingly longer. The third point is the summary of DEHP. Despite being restricted, DEHP has been the most commonly used plasticizer used in various plastic products for decades, and it is still ubiquitous [39]. In addition, the relationships between phthalate or DEHP and mortality, particularly CVD mortality, remain to be elucidated. Based on the previous literature [31], we summed the concentrations of EHHP, MEHP, MECPP, and MEOHP metabolites as the value of DEHP.

There are few studies on the mechanism of DEHP-induced CVD. Evidence from DEHP mechanistic research suggests that DEHP may cause damage to the heart, cardiac electrophysiology and vascular component cells. DEHP was injected intraperitoneally into mice for 30 days at doses of 5, 50 and 200 mg/kg body weight. Even at a DEHP dose of 5 mg/kg, DEHP resulted in an increase in inflammatory cell migration between myocardial bundles [40]. Additionally, malondialdehyde (MDA) levels, protein carbon (PC) concentrations, and DNA fragmentation were all increased in heart tissue. Increases in enzymatic (CAT) and superoxide dismutase (SOD) activities and decreases in nonenzymatic (protein bound sulfhydryl concentration (PSH)) activities and inhibited cardiac AChE activity were observed [40]. Several papillary muscle cells showed lipid droplets after oral administration of 100 mg/kg DEHP for 35 days in male mice. Further metabolomic results revealed that DEHP can inhibit fatty acid β-oxidation and gluconeogenesis, promote glycolysis and inhibit the tricarboxylic acid cycle [41]. Adult mice were implanted with radiofrequency transmitters to monitor the effects of DEHP exposure on heart rate variability and blood pressure. Mice exposed to DEHP by drinking water showed a decrease in heart rate variability and an exaggerated response to mean arterial pressure of ganglion blockade. Under stress conditions, DEHP-treated animals showed enhanced cardiovascular reactivity and prolonged blood pressure recovery time [42]. Compared with the control group, the effective refractory period of the atrioventricular node (AVN) and ventricle (VR) was increased after exposure to MEHP (a metabolite of DEHP) for 30 minutes. Additionally, epicardial conduction velocity decreased, which may be caused by the inhibition of Nav1.5 [43]. Compared to apoE^-^/^-^ mice, apoE^-^/^-^ mice treated with DEHP for 4 weeks exhibited aggravated hyperlipidemia, systemic inflammation and an atherosclerotic phenotype. DEHP promotes the oxidation of low-density lipoprotein (LDL), which leads to endotheliitis. In addition, DEHP downregulated the expression of regulatory proteins by reducing LDL receptors, cholesterol 7 α-hydrolase, ATP binding box transporters G5 and G8, and liver X receptor α and increasing the accumulation of cholesterol in the liver [44]. The vascular smooth muscle cells (VSMCs) of rats treated with 2 and 3.5 mg/L DEHP exhibited increased MMP-2 and MMP-9 activity and MMP-2 and MMP-9 protein expression. MMP-2 and MMP-9 play an important role in atherosclerosis [45]. The upstream regulatory factors p38 MAPK, ERK1/2, Akt and NF-κB of MMP-2 and MMP-9 were upregulated [46]. In male Wistar rats, the aortic rings were denuded, cultured in vitro and exposed to DEHP. Compared with the control group, the DEHP-treated group exhibited relaxation of vascular smooth muscle cells by inhibiting calcium channels [47]. These studies suggest that DEHP may cause cardiovascular damage.

Our stratified results showed that females, sedentary people, nondrinkers and people with a BMI greater than 25 were more susceptible to DEHP-related cardiovascular mortality. Regarding the increased susceptibility among women, we speculate that the first reason is that women have greater exposure to DEHP than men (data from the present study), probably because the source of DEHP exposure is related to cosmetics, and more DEHP exposure leads to a stronger effect on women. Second, DEHP may have an estrogen-regulating effect. Previous evidence suggests that DEHP can downregulate the expression of estrogen receptor α (ERα) [48, 49] and upregulate the expression of ERβ [49]. In addition, previous reports have shown that estrogen may play a role in the occurrence and development of cardiovascular diseases. Therefore, we speculated that DEHP may interfere with estrogen homeostasis and make women more susceptible to CVD mortality. We also noticed that people who do not exercise regularly were more likely to exhibit this association, and the lack of exercise itself is a risk factor related to the cause of death of CVD [50], which suggests that even under certain levels of DEHP exposure, exercise may reduce the risk of CVD death associated with DEHP. In addition, we found that nondrinkers are more likely to exhibit an association of DEHP with CVD mortality, which is very interesting and similar to the results reported in the past. It has been suggested that light and moderate alcohol intake has a protective effect against CVD mortality [51]. We speculated that alcohol consumption may lead to increased polyphenol intake, which may have a protective effect on the CVD system [52]. Finally, we found that people with a BMI greater than 25 were more likely to exhibit a relationship of DEHP with CVD mortality. Previous results also found that overweight and obesity were risk factors for CVD-induced death [53]. It is suggested that weight management may reduce the risk of death from CVD associated with DEHP. In addition, previous reports found that DEHP can cause obesity [54], which requires more research to clarify the roles and relationships of DEHP and obesity in the context of CVD mortality.

There are some limitations in the present study. First, although we found that DEHP may be a risk factor for CVD mortality in a cohort study, a longer follow-up time and larger sample size are needed for verification, and studies including people from other countries and regions are needed to support our results. Second, to avoid the interference of confounding factors, we included a series of covariates in this study, but other confounders that we were unable to assess, such as genetic factors, may have affected the results and need to be considered in future research. Third, we chose the more accurate internal exposure and DEHP concentration in urine as the internal exposure variable, but the blood DEHP concentration may reflect the in vivo exposure more accurately because the DEHP in the blood can directly contact the cells. Fourth, DEHP is metabolized rapidly in human body. Generally, it can be eliminated from the body 24 hours after ingestion. Urine DEHP at a single time point represents previous exposure. This may lead to bias due to different exposures, which may lead to different everyday DEHP exposure situations. Future research needs to assess urine data from multiple measurement time points to comprehensively evaluate DEHP exposure concentrations.

In conclusion, our results suggest that environmental DEHP exposure is an important risk factor for death in the United States, especially death from CVD. It is not surprising that DEHP exposure does not receive much attention, although DEHP is widespread throughout the environment. There are regulations or guidelines on the external environmental concentration of DEHP. For example, the maximum contaminant level of DEHP for drinking water regulated by the USEPA is 0.006 mg/L [55]. Other regulations or standards for DEHP existed, including environmental quality standards for drinking water (0.17 μg/L), environmental risk levels for soil (1 mg/kg dry weight [dw]) and sediment (1 mg/kg dw), and minor adverse effect concentrations for marine sediment (0.78 mg/kg dw) [32]. However, there are no regulations to date on the internal exposure level of DEHP. Previous studies have revealed the association between another environmental chemical (e.g., lead) and cardiovascular morbidity and mortality, and more attention should be given to the health effects of DEHP, especially the cardiovascular injury effects of DEHP. In fact, this study shows that the assessment of environmental DEHP exposure is essential for understanding CVD mortality trends and developing comprehensive strategies for the prevention of CVD.

## Supplementary Information

**Additional file 1: Figure S1.** Kaplan–Meier survival curves for MECPP and all-cause and cardiovascular disease mortality. MECPP quartiles were used, in which 4 MECPP groups were assessed for all-cause (A) and cardiovascular disease mortality (B): lowest (< 12.5 ng/mL), lower (>=12.5 to <21.1 ng/mL), higher (>=21.1 to 39.2 ng/mL) and highest (>=39.2 ng/mL) concentrations.

**Additional file 2: Figure S2.** Restricted cubic spline model of the hazard ratio and 95% confidence interval of all-cause and cardiovascular disease mortality according to urinary DEHP levels (log10 transformed) adjusted for demographic (age, sex, and race/ethnicity), lifestyle factors (education levels, poverty income ratio, alcohol consumption, smoking status, physical activity, and body mass index) and comorbidities (diabetes, hypertension, total cholesterol, alanine transaminase and high-density lipoprotein cholesterol). A. DEHP and all-cause mortality. B. DEHP and cardiovascular disease mortality.

**Additional file 3: Figure S3.** Restricted cubic spline model of the hazard ratio and 95% confidence interval of all-cause and cardiovascular disease mortality with urinary MECPP levels (log10 transformed) adjusted for demographic (age, sex, and race/ethnicity), lifestyle factors (education levels, poverty income ratio, alcohol consumption, smoking status, physical activity, and body mass index) and comorbidities (diabetes, hypertension, total cholesterol, alanine transaminase and high-density lipoprotein cholesterol). A. MECPP and all-cause mortality. B. MECPP and cardiovascular disease mortality.

**Additional file 4: Table S1.** The detection rate and distribution of urinary phthalate metabolites.

**Additional file 5: Table S2.** The association of urinary di(2-ethylhexyl) phthalate (DEHP) concentration with all-cause mortality and cardiovascular mortality from NHANES 2003–2014.

**Additional file 6: Table S3.** The association of individual urinary phthalate concentrations with all-cause mortality from NHANES 2003–2014.

**Additional file 7: Table S4.** The association of individual urinary phthalate concentrations with cardiovascular mortality from NHANES 2003–2014.

**Additional file 8: Table S5.** The urinary Di (2-ethylhexyl) phthalate (DEHP) concentration and cardiovascular mortality: indirect effect mediated by selected covariates.

## Data Availability

The dataset used and analyzed during the current study is available upon request.

## Abbreviations

CVD: Cardiovascular disease
NHANES: National Health and Nutrition Examination Survey
HR: Hazard ratio
CI: Confidence interval
DEHP: di(2-ethylhexyl) phthalate
MECPP: Mono-2-ethyl-5-carboxypentyl phthalate
PM_2.5_: Fine particulate matter
BPA: Bisphenol A
DOP: Dioctyl phthalate
BEHP: Dioctyl phthalate (2-ethylhexyl)
MEHP: Mono(2-ethylhexyl) phthalate
RCS: Restricted cubic spline
CDC: Centers for Disease Control
MCNP: Mono(carboxynonyl) phthalate
MCOP: Mono(carboxyoctyl) phthalate
MnBP: Mono-n-butyl phthalate
MCPP: Mono-(3-carboxypropyl) phthalate
MEP: Mono-ethyl phthalate
MEHHP: Mono-(2-ethyl-5-hydroxyhexyl) phthalate
MiBP: Mono-isobutyl phthalate
MiNP: Mono-isononyl phthalate
MEOHP: Mono-(2-ethyl-5-oxohexyl) phthalate
ICD-10: International Classification of Diseases 10th Revision
PIR: Poverty income ratio
BMI: Body mass index
ALT: Alanine transaminase
HDLC: High-density lipoprotein cholesterol
MDA: Malondialdehyde
PC: Protein carbon
CAT: Enzymatic
SOD: Superoxide dismutase
PSH: Protein bound sulfhydryl
AVN: Atrioventricular node
VR: Ventricle
LDL: Low-density lipoprotein
VSMC: Vascular smooth muscle cell
ER: Estrogen receptor
