# Supplementary figures and images for: Low-level plasticizer exposure and all-cause and cardiovascular disease mortality in the general population
Source: Environ Health. 2022 Mar 9;21:32. doi: 10.1186/s12940-022-00841-3 (PMC8905760; doi:10.1186/s12940-022-00841-3)

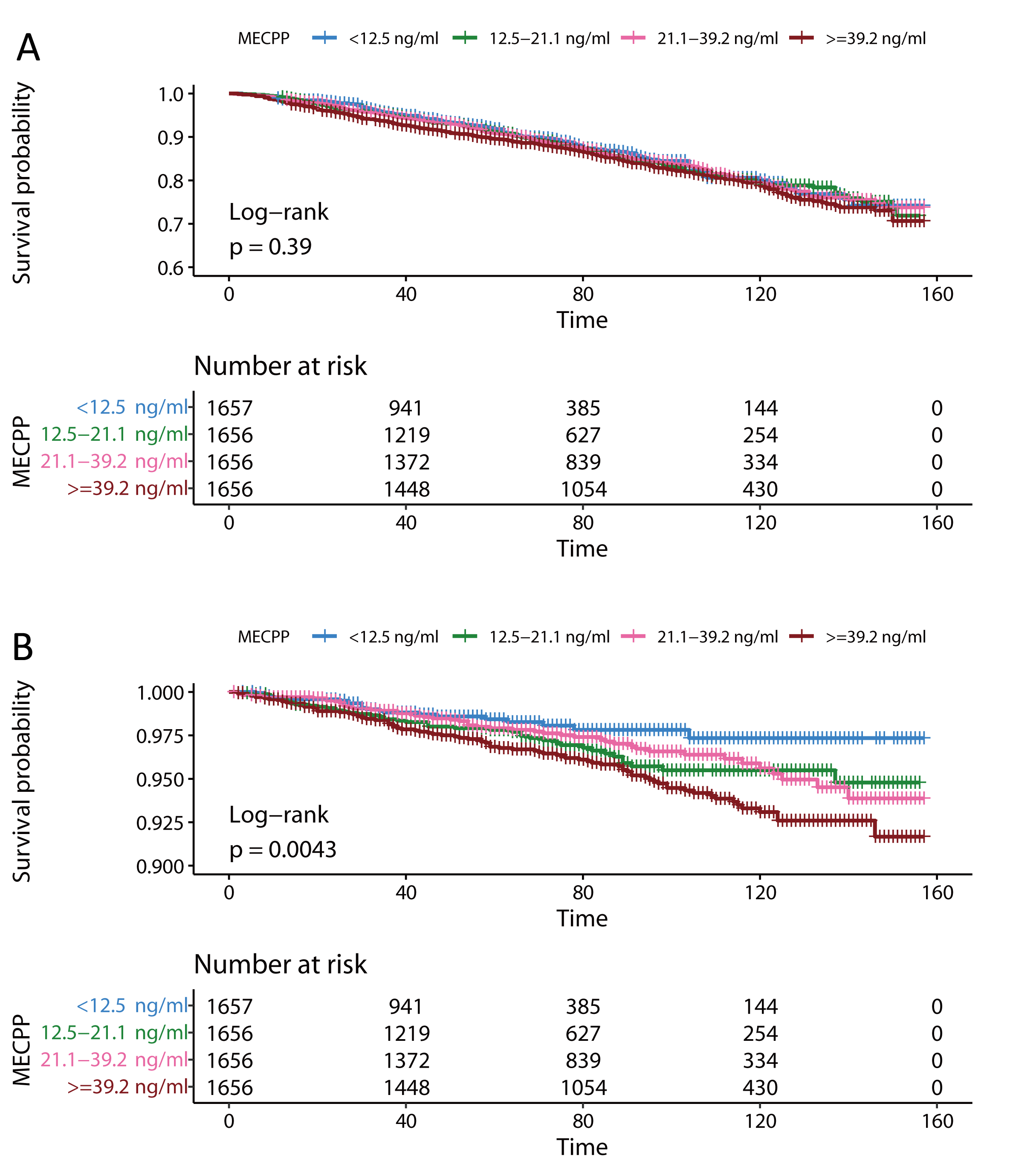

Supplement: Supplementary file 1 — Additional file 1: Figure S1. Kaplan–Meier survival curves for MECPP and all-cause and cardiovascular disease mortality. MECPP quartiles were used, in which 4 MECPP groups were assessed for all-cause (A) and cardiovascular disease mortality (B): lowest (< 12.5 ng/mL), lower (>=12.5 to <21.1 ng/mL), higher (>=21.1 to 39.2 ng/mL) and highest (>=39.2 ng/mL) concentrations. [file 12940_2022_841_MOESM1_ESM.tif]

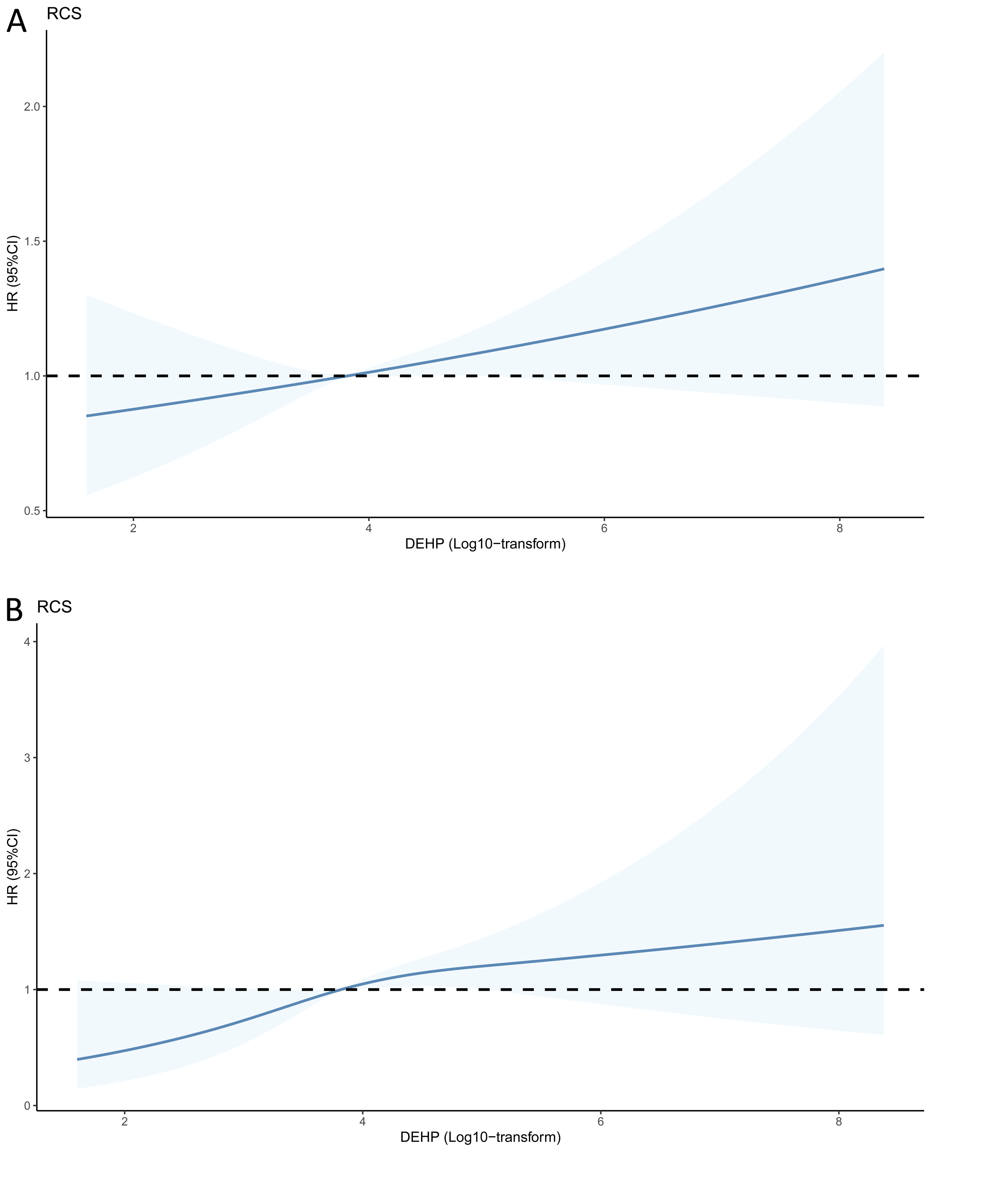

Supplement: Supplementary file 2 — Additional file 2: Figure S2. Restricted cubic spline model of the hazard ratio and 95% confidence interval of all-cause and cardiovascular disease mortality according to urinary DEHP levels (log10 transformed) adjusted for demographic (age, sex, and race/ethnicity), lifestyle factors (education levels, poverty income ratio, alcohol consumption, smoking status, physical activity, and body mass index) and comorbidities (diabetes, hypertension, total cholesterol, alanine transaminase and high-density lipoprotein cholesterol). A. DEHP and all-cause mortality. B. DEHP and cardiovascular disease mortality. [file 12940_2022_841_MOESM2_ESM.tif]

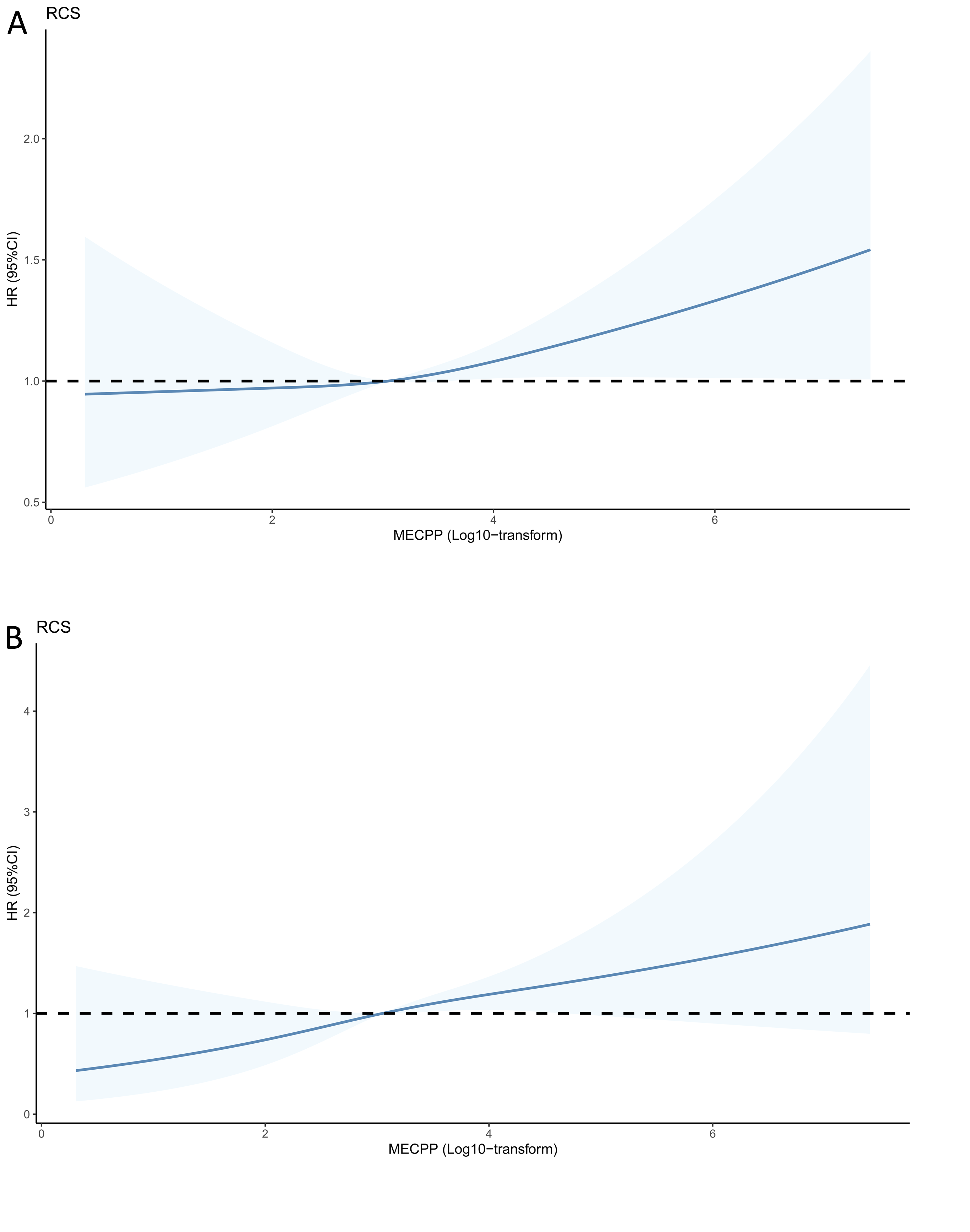

Supplement: Supplementary file 3 — Additional file 3: Figure S3. Restricted cubic spline model of the hazard ratio and 95% confidence interval of all-cause and cardiovascular disease mortality with urinary MECPP levels (log10 transformed) adjusted for demographic (age, sex, and race/ethnicity), lifestyle factors (education levels, poverty income ratio, alcohol consumption, smoking status, physical activity, and body mass index) and comorbidities (diabetes, hypertension, total cholesterol, alanine transaminase and high-density lipoprotein cholesterol). A. MECPP and all-cause mortality. B. MECPP and cardiovascular disease mortality. [file 12940_2022_841_MOESM3_ESM.tif]
