# Supplementary material for: Low-level plasticizer exposure and all-cause and cardiovascular disease mortality in the general population
Source: Environ Health. 2022 Mar 9;21:32. doi: 10.1186/s12940-022-00841-3 (PMC8905760; doi:10.1186/s12940-022-00841-3)
Supplement: Supplementary file 4 — Additional file 4: Table S1. The detection rate and distribution of urinary phthalate metabolites. [file 12940_2022_841_MOESM4_ESM.docx]

**Table S1.** The detection rate and distribution of urinary phthalates metabolites

|  |  |  |  | Urinary phthalates concentration (ng/ml) | | | | |
| --- | --- | --- | --- | --- | --- | --- | --- | --- |
| Phthalates name | Abbreviation | N | Detection rate (%) | 10% | 25% | median | 75% | 90% |
| Mono(carboxynonyl) phthalate | MCNP | 5650 | 94.80 | 0.71 | 1.52 | 2.54 | 4.73 | 9.13 |
| Mono(carboxyoctyl) phthalate | MCOP | 5650 | 97.88 | 1.94 | 4.51 | 9.71 | 25.73 | 67.32 |
| Mono-2-ethyl-5-carboxypentyl phthalate | MECPP | 6625 | 99.85 | 7.67 | 12.46 | 21.05 | 39.22 | 79.64 |
| Mono-n-butyl phthalate | MnBP | 8591 | 98.26 | 5.65 | 9.68 | 16.38 | 27.54 | 46.71 |
| Mono-(3-carboxypropyl) phthalate | MCPP | 7670 | 95.17 | 0.84 | 1.43 | 2.51 | 4.53 | 9.23 |
| Mono-ethyl phthalate | MEP | 8586 | 99.88 | 11.91 | 34.14 | 86.18 | 268.76 | 804.85 |
| Mono-(2-ethyl-5-hydroxyhexyl) phthalate | MEHHP | 7670 | 99.49 | 4.38 | 7.54 | 13.68 | 25.87 | 54.66 |
| Mono-(2-ethyl)-hexyl phthalate | MEHP | 8591 | 63.61 | 0.56 | 0.92 | 1.78 | 3.57 | 7.55 |
| Mono-isobutyl pthalate | MiBP | 7670 | 97.52 | 2.13 | 3.94 | 7.06 | 12.15 | 20.42 |
| Mono-isononyl phthalate | MiNP | 8591 | 23.46 | 0.43 | 0.63 | 1.02 | 1.99 | 4.06 |
| Mono-(2-ethyl-5-oxohexyl) phthalate | MEOHP | 7670 | 98.88 | 2.90 | 4.80 | 8.38 | 15.44 | 32.02 |
| Mono-benzyl phthalate | MBzP | 8591 | 98.02 | 1.72 | 3.22 | 6.09 | 11.67 | 21.92 |
