# Supplementary material for: Low-level plasticizer exposure and all-cause and cardiovascular disease mortality in the general population
Source: Environ Health. 2022 Mar 9;21:32. doi: 10.1186/s12940-022-00841-3 (PMC8905760; doi:10.1186/s12940-022-00841-3)
Supplement: Supplementary file 5 — Additional file 5: Table S2. The association of urinary di(2-ethylhexyl) phthalate (DEHP) concentration with all-cause mortality and cardiovascular mortality from NHANES 2003–2014. [file 12940_2022_841_MOESM5_ESM.docx]

**Table S2.** The association of urinary Di (2-ethylhexyl) phthalate (DEHP) concentration with all-cause mortality and cardiovascular mortality in NHANES 2003–2014

|  | Urinary DEHP levels (mg/g) | | | |  |
| --- | --- | --- | --- | --- | --- |
|  | <2.38 | 2.38-4.03 | 4.03-7.38 | ≥7.38 | P for trend |
| All-cause mortality |  |  |  |  |  |
| Crude | Ref | 1.23 (0.98, 1.54) | 1.35 (1.09, 1.68) | 1.48 (1.20, 1.83) | <0.001 |
| Model 1 | Ref | 1.09 (0.87, 1.37) | 1.13 (0.91, 1.41) | 1.30 (1.05, 1.62) | 0.009 |
| Model 2 | Ref | 1.06 (0.85, 1.34) | 1.10 (0.88, 1.37) | **1.25 (1.01, 1.55)** | 0.024 |
| CVD mortality |  |  |  |  |  |
| Crude | Ref | 1.97 (1.12, 3.45) | 2.42 (1.41, 4.15) | 2.87 (1.70, 4.86) | <0.001 |
| Model 1 | Ref | 1.76 (1.00, 3.09) | 2.05 (1.19, 3.53) | 2.59 (1.52, 4.41) | <0.001 |
| Model 2 | Ref | 1.75 (0.99, 3.08) | **2.01 (1.16, 3.48)** | **2.54 (1.49, 4.32)** | <0.001 |

Values are hazard ratio (95% confidence interval).

Crude was not adjusted.

Model 1: adjusted for age (years, continuous), sex (female or male), and race/ethnicity (non-Hispanic white, black, Hispanic-Mexican, or other).

Mode 2 : model 1 plus adjusted for education levels (Less Than 9th Grade, 9-11th Grade, High School Grad/GED or Equivalent, Some College or AA degree, College Graduate or above), poverty to income ratio (<1, ≥1, or missing), physical activity (never, moderate, vigorous or missing), smoking status (never, ever or current), past-year alcohol drinking (no, yes, or missing), body mass index (<25, 25–30, or ≥30 kg/m^2^), total cholesterol (mg/dL, continuous), alanine aminotransferase (U/L, continuous), high-density lipoprotein cholesterol (mg/dL, continuous), hypertension (no/yes), diabetes (no/yes).
