# Supplementary material for: Low-level plasticizer exposure and all-cause and cardiovascular disease mortality in the general population
Source: Environ Health. 2022 Mar 9;21:32. doi: 10.1186/s12940-022-00841-3 (PMC8905760; doi:10.1186/s12940-022-00841-3)
Supplement: Supplementary file 6 — Additional file 6: Table S3. The association of individual urinary phthalate concentrations with all-cause mortality from NHANES 2003–2014. [file 12940_2022_841_MOESM6_ESM.docx]

**Table S3.** The association of individual urinary phthalate concentrations with all-cause mortality in NHANES 2003–2014

|  | Q1 | Q2 | Q3 | Q4 | P for trend |
| --- | --- | --- | --- | --- | --- |
| MCNP | Ref | 0.99 (0.80, 1.24) | 0.97 (0.77, 1.23) | 0.99 (0.78, 1.26) | 0.839 |
| MCOP | Ref | 1.01 (0.82, 1.25) | 1.04 (0.83, 1.30) | 1.06 (0.83, 1.37) | 0.374 |
| MECPP | Ref | 1.07 (0.86, 1.33) | 1.05 (0.85, 1.29) | 1.30 (1.06, 1.60) | 0.042* |
| MnBP | Ref | 1.23 (1.00, 1.52) | 1.34 (1.10, 1.64) | 1.41 (1.15, 1.74) | 0.359 |
| MCPP | Ref | 0.89 (0.73, 1.09) | 1.16 (0.96, 1.40) | 1.26 (1.04, 1.53) | 0.254 |
| MEP | Ref | 0.96 (0.80, 1.17) | 1.00 (0.83, 1.21) | 0.95 (0.78, 1.16)  1.31 (1.07, 1.60) | 0.312 |
| MEHHP | Ref | 1.09 (0.89, 1.34) | 1.07 (0.88, 1.32) |  | 0.386 |
| MEHP | Ref | 1.08 (0.90, 1.29) | 0.89 (0.73, 1.08) | 1.08 (0.90, 1.31) | 0.274 |
| MiBP | Ref | 1.08 (0.91, 1.28) | 0.95 (0.78, 1.16) | 1.12 (0.91, 1.37) | 0.156 |
| MiNP | Ref | 0.84 (0.70, 1.01) | 0.96 (0.80, 1.15) | 0.97 (0.80, 1.19) | 0.163 |
| MEOHP | Ref | 0.93 (0.75, 1.15) | 1.04 (0.85, 1.27) | 1.16 (0.95, 1.42) | 0.265 |
| MBzP | Ref | 1.13 (0.92, 1.39) | 1.24 (1.02, 1.52) | 1.47 (1.21, 1.79) | <0.001* |

Q, quartile.

Mono(carboxynonyl) phthalate (MCNP), mono(carboxyoctyl) phthalate (MCOP), mono-2-ethyl-5-carboxypentyl phthalate (MECPP), mono-n-butyl phthalate (MnBP), mono-(3-carboxypropyl) phthalate (MCPP), mono-ethyl phthalate (MEP), mono-(2-ethyl-5-hydroxyhexyl) phthalate (MEHHP), mono-(2-ethyl)-hexyl phthalate (MEHP), mono-isobutyl pthalate (MiBP), mono-isononyl phthalate (MiNP), and mono-(2-ethyl-5-oxohexyl) phthalate (MEOHP).

Values are hazard ratio (95% confidence interval).

Adjusted for age (years, continuous), sex (female or male), and race/ethnicity (non-Hispanic white, black, Hispanic-Mexican, or other), education levels (Less Than 9th Grade, 9-11th Grade, High School Grad/GED or Equivalent, Some College or AA degree, College Graduate or above), poverty to income ratio (<1, ≥1, or missing), physical activity (never, moderate, vigorous or missing), smoking status (never, ever or current), past-year alcohol drinking (no, yes, or missing), body mass index (<25, 25–30, or ≥30 kg/m^2^), total cholesterol (mg/dL, continuous), alanine aminotransferase (U/L, continuous), high-density lipoprotein cholesterol (mg/dL, continuous), hypertension (no/yes), diabetes (no/yes).

*, after Holm-Bonferroni correction, p value < (0.05/n=12)
