# Supplementary material for: Low-level plasticizer exposure and all-cause and cardiovascular disease mortality in the general population
Source: Environ Health. 2022 Mar 9;21:32. doi: 10.1186/s12940-022-00841-3 (PMC8905760; doi:10.1186/s12940-022-00841-3)
Supplement: Supplementary file 7 — Additional file 7: Table S4. The association of individual urinary phthalate concentrations with cardiovascular mortality from NHANES 2003–2014. [file 12940_2022_841_MOESM7_ESM.docx]

**Table S4.** The association of individual urinary phthalate concentrations with cardiovascular mortality in NHANES 2003–2014

|  | Q1 | Q2 | Q3 | Q4 | P for trend |
| --- | --- | --- | --- | --- | --- |
| MCNP | Ref | 1.02 (0.61, 1.70) | 1.06 (0.63, 1.80) | 1.12 (0.66, 1.92) | 0.650 |
| MCOP | Ref | 0.93 (0.59, 1.48) | 0.69 (0.40, 1.18) | 0.95 (0.55, 1.63) | 0.501 |
| MECPP | Ref | 1.53 (0.93, 2.54) | 1.37 (0.83, 2.27) | **2.33 (1.45, 3.73)*** | <0.001 |
| MnBP | Ref | 1.42 (0.90, 2.26) | 1.63 (1.04, 2.55) | 1.89 (1.21, 2.96) | 0.004 |
| MCPP | Ref | 0.95 (0.64, 1.43) | 0.97 (0.64, 1.46) | 1.20 (0.80, 1.80) | 0.364 |
| MEP | Ref | 0.99 (0.68, 1.46) | 0.86 (0.58, 1.27) | 0.83 (0.55, 1.26) | 0.288 |
| MEHHP | Ref | 1.47 (0.92, 2.34) | 1.44 (0.91, 2.29) | 1.89 (1.20, 2.96) | 0.009 |
| MEHP | Ref | 1.29 (0.88, 1.90) | 1.11 (0.74, 1.68) | 1.35 (0.89, 2.03) | 0.267 |
| MiBP | Ref | 0.93 (0.64, 1.33) | 0.97 (0.65, 1.44) | 0.81 (0.52, 1.28) | 0.451 |
| MiNP | Ref | 0.96 (0.63, 1.46) | 1.30 (0.87, 1.93) | 1.54 (1.02, 2.32) | 0.015 |
| MEOHP | Ref | 1.29 (0.78, 2.13) | 1.70 (1.06, 2.72) | 1.90 (1.19, 3.03) | 0.003 |
| MBzP | Ref | 0.94 (0.61, 1.45) | 1.11 (0.74, 1.68) | 1.30 (0.86, 1.95) | 0.129 |

Q, quartile.

Mono(carboxynonyl) phthalate (MCNP), mono(carboxyoctyl) phthalate (MCOP), mono-2-ethyl-5-carboxypentyl phthalate (MECPP), mono-n-butyl phthalate (MnBP), mono-(3-carboxypropyl) phthalate (MCPP), mono-ethyl phthalate (MEP), mono-(2-ethyl-5-hydroxyhexyl) phthalate (MEHHP), mono-(2-ethyl)-hexyl phthalate (MEHP), mono-isobutyl pthalate (MiBP), mono-isononyl phthalate (MiNP), and mono-(2-ethyl-5-oxohexyl) phthalate (MEOHP).

Values are hazard ratio (95% confidence interval).

Adjusted for age (years, continuous), sex (female or male), and race/ethnicity (non-Hispanic white, black, Hispanic-Mexican, or other), education levels (Less Than 9th Grade, 9-11th Grade, High School Grad/GED or Equivalent, Some College or AA degree, College Graduate or above), poverty to income ratio (<1, ≥1, or missing), physical activity (never, moderate, vigorous or missing), smoking status (never, ever or current), past-year alcohol drinking (no, yes, or missing), body mass index (<25, 25–30, or ≥30 kg/m^2^), total cholesterol (mg/dL, continuous), alanine aminotransferase (U/L, continuous), high-density lipoprotein cholesterol (mg/dL, continuous), hypertension (no/yes), diabetes (no/yes).

*, after Holm-Bonferroni correction, p value < (0.05/n=12)
