# Supplementary material for: Low-level plasticizer exposure and all-cause and cardiovascular disease mortality in the general population
Source: Environ Health. 2022 Mar 9;21:32. doi: 10.1186/s12940-022-00841-3 (PMC8905760; doi:10.1186/s12940-022-00841-3)
Supplement: Supplementary file 8 — Additional file 8: Table S5. The urinary Di (2-ethylhexyl) phthalate (DEHP) concentration and cardiovascular mortality: indirect effect mediated by selected covariates. [file 12940_2022_841_MOESM8_ESM.docx]

**Table S5.** The urinary Di (2-ethylhexyl) phthalate (DEHP) concentration and cardiovascular mortality: indirect effect mediated by selected covariates

| Covariates | Proportion Mediated, % | P Value for  Estimated Indirect Effect |
| --- | --- | --- |
| Sex | 1.91 | 0.001 |
| Alcohol drinking status | -0.17 | 0.077 |
| BMI | 2.43 | <0.001 |
| Physical activity | 2.82 | <0.001 |

Adjusted for age (years, continuous), sex (female or male), race/ethnicity (non-Hispanic white, black, Hispanic-Mexican, or other), education levels (Less Than 9th Grade, 9-11th Grade, High School Grad/GED or Equivalent, Some College or AA degree, College Graduate or above), poverty to income ratio (<1, ≥1, or missing), physical activity (never, moderate, vigorous or missing), smoking status (never, ever or current), past-year alcohol drinking (no, yes, or missing), body mass index (<25, 25–30, or ≥30 kg/m2), total cholesterol (mg/dL, continuous), alanine aminotransferase (U/L, continuous), high-density lipoprotein cholesterol (mg/dL, continuous), hypertension (no/yes), diabetes (no/yes).
